# Chrysophanol Attenuates Manifestations of Immune Bowel Diseases by Regulation of Colorectal Cells and T Cells Activation In Vivo

**DOI:** 10.3390/molecules26061682

**Published:** 2021-03-17

**Authors:** Hyun-Su Lee, Gil-Saeng Jeong

**Affiliations:** College of Pharmacy, Keimyung University, Daegu 42601, Korea; hyunsu.lee@kmu.ac.kr

**Keywords:** colorectal cells, HT-29, colitis, chrysophanol, pro-inflammatory cytokines, effector T cells, DSS-induced IBD, immunosuppressive

## Abstract

Inflammatory bowel disease (IBD) is an immune disorder that develops due to chronic inflammation in several cells. It is known that colorectal and T cells are mainly involved in the pathogenesis of IBD. Chrysophanol is an anthraquinone family member that possesses several bioactivities, including anti-diabetic, anti-tumor, and inhibitory effects on T cell activation. However, it is unknown whether chrysophanol suppresses the activity of colorectal cells. In this study, we found that chrysophanol did not induce cytotoxicity in HT-29 colorectal cells. Pre-treatment with chrysophanol inhibited the mRNA levels of pro-inflammatory cytokines in tumor necrosis factor-α (TNF-α)-stimulated HT-29 cells. Western blot analysis revealed that pre-treatment with chrysophanol mitigates p65 translocation and the mitogen-activated protein kinase (MAPK) pathway in activated HT-29 cells. Results from the in vivo experiment confirmed that oral administration of chrysophanol protects mice from dextran sulfate sodium (DSS)-induced IBD. Chrysophanol administration attenuates the expression of pro-inflammatory cytokines in colon tissues of the DSS-induced IBD model. In addition, we found that oral administration of chrysophanol systemically decreased the expression of effector cytokines from mesenteric lymph nodes. Therefore, these data suggest that chrysophanol has a potent modulatory effect on colorectal cells as well as exhibiting a beneficial potential for curing IBD in vivo.

## 1. Introduction

Inflammatory bowel disease (IBD) is an immunologically associated disease that mainly occurs in the colon [1]. It has been reported to be caused by uncontrolled inflammatory responses due to the continuous but improper activity of immune cells, unsuitable diet and stress [2]. IBD is classified into two by cause of disease, Crohn’s disease (CD) and Ulcerative colitis (UC). CD mainly develops inflammation in the overall digestive tract, which has a deep impact on colonic tissues; however, UC occurs in the rectum and colon in particular [3]. It also has been studied that IBD mostly affects both young and elderly adults but most first diagnosis of IBD is almost before 30 years old [4]. Nevertheless, CD and UC exhibit the most general forms of IBD that are categorized by environmental and genetic factors; however, the exact mechanism and cause of IBD pathogenesis remain elusive [5]. Recent pharmaceutical trials to overcome IBD have included (1) four proprietary antibody therapies against tumor necrosis factor-α (TNF-α) (adalimumab, golimumab, infliximab, certolizumab), (2) inhibitor for Janus kinase (tofacitinib), (3) inhibitor for α4β7 integrin (vedolizumab) and (4) inhibitor for interleukin-12/23 (IL-12/IL-23) cytokine (ustekinumab) [6]. Although existing therapies in the market for IBD are mostly based on blocking the pro-inflammatory factors, there have been few trials using natural product-derived bioactive small molecules that fundamentally affect cell activation in IBD pathology.

Several clinical manifestations of IBD have been reported to include drastic loss of body weight, bloody excrement, diarrhea, destroyed structure of colon tissue, and swelling of lymph nodes [7]. Continuous inflammatory responses include the production of pro-inflammatory cytokines, including TNF-α, interleukin 1-β (IL-1β), and interleukin 8 (IL-8) in colon tissues [8]. Therefore, chronic inflammation in activated colon tissues involves the activity of effector T helper type 1 and 17 (Th1 and Th17) cells that release interferon-γ (IFN-γ) and interleukin 17 (IL-17), which play a critical role in the pathogenesis of IBD [9]. Despite tremendous efforts to develop therapeutics for IBD using small bioactive molecules, little progress has been reported.

Chrysophanol (C_15_H_10_O_4_), a bioactive molecule in the anthraquinone family, has been reported to be isolated from various natural products such as *Rumex crispus L*, *Cluytia hirsute*, *Rheum palmatum*, *Aloe excels*, and *Cassia fistula* [10,11,12,13,14]. Several bioactivities of chrysophanol have been elucidated, including a protective effect on learning and memory function in Alzheimer’s disease, antitumor, and anti-diabetes [15,16,17]. In particular, the anti-inflammatory effect of chrysophanol has been investigated that efficiently regulates lipopolysaccharide (LPS)-induced inflammation in RAW264.7 cells through nuclear factor kappa-light chain enhancer of activated B cells (NFκB) and peroxisome proliferator-activated receptor γ (PPARγ) pathway [18]. Another literature has shown that chrysophanol has an anti-atopic effect via the mitogen-activated protein kinase (MAPK) signaling pathway in activated HMC-1 mast cell lines [19]. Recently, our previous publication demonstrated the modulatory effect of chrysophanol on T cell activation, which is the most important factor for colitis and intestinal inflammation [20,21]. Although the suppressive effects of chrysophanol on the activity of peritoneal macrophages have been investigated [22], little is known about the inhibitory effect of chrysophanol on colorectal and T cells.

In the current study, we elucidated whether chrysophanol has a regulatory effect on the activity of colorectal cells in vitro without cytotoxicity. To examine the therapeutic potential of chrysophanol in inflammatory conditions in vivo, a dextran sulfate sodium (DSS)-induced IBD model was used as an animal model and dose-dependent administration was performed. The reduced activity of effector T cells following the administration of chrysophanol was also confirmed.

## 2. Results

### 2.1. Chrysophanol Does Not Show Cytotoxicity on HT-29 Colorectal Cells

**Figure 1:**
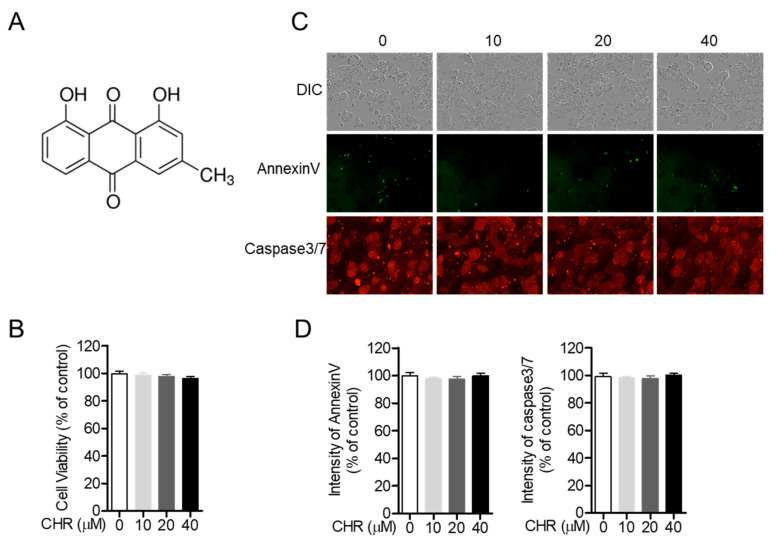
Chrysophanol (CHR) does not show cytotoxicity on HT-29 colorectal cells. (**A**) The chemical structure of chrysophanol. (**B**) HT-29 cells (1 × 10^4^/well, 96-well plate) were treated with the indicated concentrations of chrysophanol (0 to 40 μM) for 24 h and methyl thiazol tetrazolium (MTT) assay was performed for determination of cell viability. (**C**,**D**) HT-29 cells (1 × 10^4^/well, 96-well plate) were treated with the indicated concentration (0 to 40 μM) of chrysophanol for 24 h and the intensity of AnnexinV and Caspase3/7 was assessed by IncuCyte imaging system. Obtained cell images (**C**) and intensity of AnnexinV or Caspase3/7 is presented in bar graph (% of control). DIC; differential interference contrast. (**D**) The mean value of three experiments ±SEM is presented. SEM; standard error of the mean.

Since previous studies have demonstrated the cytotoxicity of chrysophanol in several cell lines [20,23,24], we confirmed whether treatment with chrysophanol causes cell damage in HT-29 colorectal cells. Figure 1B shows that treatment with chrysophanol up to 40 μM for 24 h did not induce cell death. Differential interference contrast (DIC) images of HT-29 cells treated with chrysophanol for 24 h revealed no morphological alteration (Figure 1C, upper panel). To investigate whether treatment with chrysophanol was associated with the apoptotic pathway in HT-29 cells, the expression of AnnexinV and Caspase3/7 was assessed using the IncuCyte imaging system. Figure 1C,D shows that the intensity of AnnexinV and Caspase3/7 was independent of the concentration of chrysophanol. These results clearly suggest that treatment with chrysophanol is not associated with cytotoxicity in HT-29 cells.

### 2.2. Pre-Treatment with Chrysophanol Inhibits the Expression of Pro-Inflammatory Cytokines in Stimulated HT-29 Cells

**Figure 2:**
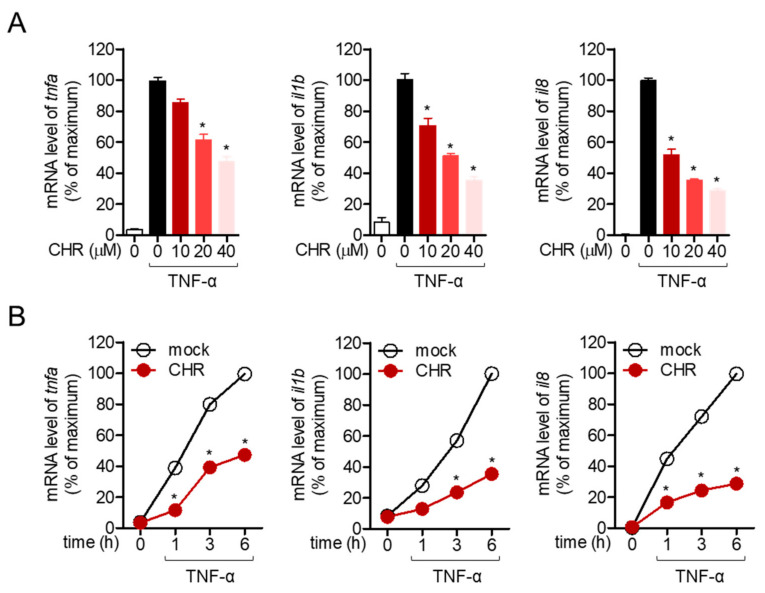
Pre-treatment with chrysophanol inhibits the expression of pro-inflammatory cytokines in stimulated HT-29 cells. (**A**) HT-29 cells pre-treated with the indicated concentrations (0 to 40 μM) of chrysophanol for 1 h were stimulated with tumor necrosis factor-α (TNF-α) (10 ng/mL) for 6 h and harvested for the isolation of mRNA. The mRNA levels of indicated genes are presented in bar graph. (**B**) HT-29 cells pre-treated with 40 μM of chrysophanol for 1 h were stimulated with TNF-α (10 ng/mL) for the indicated time (0 to 6 h). Collected cells were lysed for mRNA isolation and the mRNA levels of the indicated genes are presented in line graph. The mean value of three experiments ±SEM is presented. * *p* < 0.05 compared with TNF-α-treated cells (**A**) or mock-treated cells (**B**). IL-1b; interleukin-1 beta, IL-8: interleukin-8.

Stimulation of colonic cells with TNF-α in vitro is a widely established method to mimic the inflammatory situation in the colon. To explore whether pre-treatment with chrysophanol ameliorates the inflammatory response in TNF-α stimulation in HT-29 colonic cells, the mRNA levels of pro-inflammatory genes were determined by quantitative polymerase chain reaction (PCR) analysis. Representative pro-inflammatory genes in colonic cells, including TNF-α, IL-1β, and IL-8, were monitored. Figure 2A shows that dose-dependent pre-treatment with chrysophanol up to 40 μM suppressed the mRNA levels of pro-inflammatory genes in TNF-α stimulated conditions. In addition, time-dependent experiment with 40 μM chrysophanol was performed and it was observed that pre-treatment with chrysophanol clearly inhibits the colonic inflammation in the stimulation with TNF-α (Figure 2B). These quantitative PCR results demonstrated that chrysophanol reduced the expression of pro-inflammatory genes in colonic cells.

### 2.3. Pre-Treatment with Chrysophanol Mitigates p65 Translocation and MAPK Pathway in Activated HT-29 Cells

**Figure 3:**
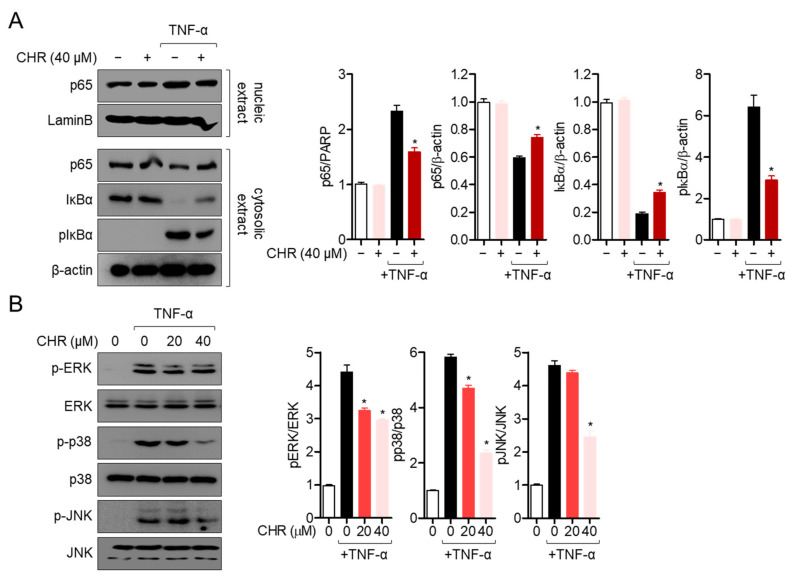
Pre-treatment with chrysophanol mitigates p65 translocation and the mitogen-activated protein kinase (MAPK) pathway in activated HT-29 cells. (**A**) Starved HT-29 cells pre-treated with 40 μM of chrysophanol for 1 h were stimulated with 10 ng/mL of TNF-α for 1 h. Nucleic extract was separated from whole lysate by using a NE-PER kit. The indicated proteins were detected and normalized with the levels of LaminB (nucleic extract) or β–actin (cytosolic extract). (**B**) Starved HT-29 cells pre-treated with the indicated concentration of chrysophanol for 1 h were stimulated with 10 ng/mL of TNF-α for 30 min. The phosphorylation levels of the indicated proteins were detected and normalized with the levels of total proteins indicated. The mean value of three experiments ±SEM is presented. * *p* < 0.05 compared with TNF-α-treated cells. IκBα; inhibitor of NF-κB, pIκBα; phosphorylated inhibitor of NF-κB, p-ERK; phosphorylated extracellular signal-related kinase, ERK; extracellular signal-related kinase, p-JNK; phosphorylated c-Jun N-terminal kinase, JNK; c-Jun N-terminal kinase.

The underlying mechanisms of the ameliorative effect of chrysophanol on TNF-α stimulated colonic inflammation were elucidated. Since the NF-κB pathway has been shown to play an essential role in the colonic inflammatory response, p65 translocation into the nucleus was determined by Western blot assay. As shown in Figure 3A, cytosolic p65 transmigrated into the nucleus after stimulation with TNF-α, but pre-treatment with chrysophanol blocked the transmigration of p65. In addition, the degradation and phosphorylation levels of IκBα were also explored in TNF-α stimulation. Degradation and phosphorylation of inhibitor of NF-κB (IκB) by TNF-α stimulation were significantly suppressed by pre-treatment with chrysophanol (Figure 3A). To investigate whether the suppressive effect of chrysophanol on colonic inflammation is associated with the reduction of the MAP kinase pathway in HT-29 cells, the phosphorylation levels of extracellular signal-regulated kinase (ERK), p38, and c-Jun N-terminal kinase (JNK) were assessed by Western blot assay. Figure 3B clearly shows that phosphorylated ERK, p38, and JNK were significantly downregulated by pre-treatment with chrysophanol in a dose-dependent manner in HT-29 colonic cells. These data suggest that chrysophanol has a regulatory effect on TNF-α-stimulated inflammation via the NF-κB and MAP kinase pathways in HT-29 colonic cells.

### 2.4. Oral Administration of Chrysophanol Protects Mice from DSS-Induced IBD In Vivo

**Figure 4:**
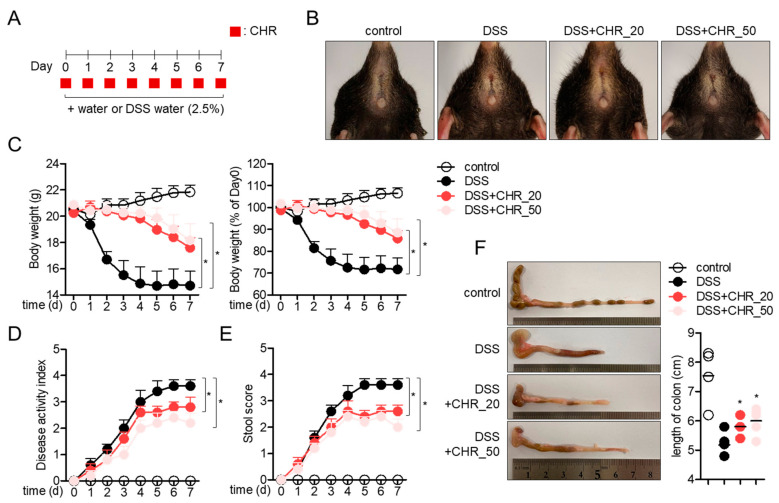
Oral administration of chrysophanol protects mice from DSS-induced IBD in vivo. (**A**) The scheme of IBD experiment using 2.5% DSS-containing water and oral administration of chrysophanol for 7 days. (**B**) The representative picture of mice anus at 7-day post exposure to 2.5% DSS-containing water and oral administration of chrysophanol. Control mice group treated with fresh drinking water, DSS mice group treated with drinking water containing 2.5% DSS for 7 days, DSS + CHR_20 mice group treated with drinking water containing 2.5% DSS and daily orally administered 20 mg/kg chrysophanol for 7 days, and DSS + CHR_50 mice group treated with drinking water containing 2.5% DSS and daily orally administered 50 mg/kg chrysophanol for 7 days (*n* = 5 mice/group). (**C**) The changes of mice body weight during exposure to DSS-containing water and oral administration of chrysophanol for 7 days (left) and the ratio of mice body weight (% of Day 0) (right). (**D**) The changes of disease activity index during exposure to DSS-containing water and oral administration of chrysophanol for 7 days. (**E**) The changes of mice stool during exposure to DSS-containing water and administration of chrysophanol for 7 days. (**F**) The representative pictures of mice colons at 7-day post exposure to DSS-containing water and oral administration of chrysophanol. The mean value of five mice ±SEM is presented. * *p* < 0.05 compared with DSS-treated group.

To elucidate whether the suppressive effect of chrysophanol on colonic inflammation is mediated by attenuating potential in an animal model, a DSS-induced IBD model using mice was used. Colonic inflammation was induced by administration of 2.5% DSS for 7 days, and two doses of chrysophanol were orally administered every day (Figure 4A). The representative images of mice anus from each group showed that administration of 2.5% DSS caused severe inflammation through the colon and anus, but simultaneous administration of chrysophanol significantly attenuated the inflammatory manifestation in a dose-dependent manner. The alteration of body weights from experimental mice revealed that the administration of chrysophanol protects mice from DSS-induced colonic inflammation (Figure 4C). Dose-dependent reduction in the disease activity index was assessed in DSS mice administered with chrysophanol (Figure 4D). Correlated stool scores also showed that oral administration of chrysophanol mitigated inflammatory manifestation (Figure 4E). Presented colons isolated from each mouse group clearly revealed that oral administration of chrysophanol blocked colon shrinkage induced by DSS intake (Figure 4F). These data suggest that oral administration of chrysophanol protects mice from DSS-induced IBD in vivo.

### 2.5. Oral Administration of Chrysophanol Attenuates the Expression of Pro-Inflammatory Cytokines on Colon Tissues of DSS-Induced IBD Model

**Figure 5:**
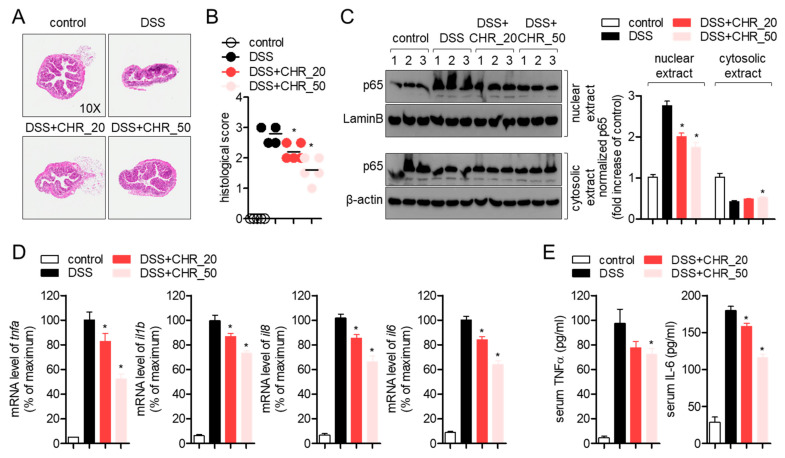
Oral administration of chrysophanol attenuates the expression of pro-inflammatory cytokines on colon tissues of the DSS-induced IBD model. (**A**) The representative images of hematoxylin and eosin (H&E) staining sections from each group. (**B**) The histological scores of H&E staining sections from each group. (**C**) Isolated colon tissues from each mouse were collected and nuclear extracts were separated from whole lysate by using the NE-PER kit. After separation, nuclear extracts and cytosolic extracts were loaded onto sodium dodecyl sulfate-polyacrylamide electrophoresis (SDS-PAGE) gels and the expression of p65 was detected. The expression of LaminB and β-actin was used for normalization in nuclear extract and cytosolic extract, respectively. (**D**) The mRNA levels of the indicated genes from colonic tissues were normalized with the levels of *GAPDH* and presented in percentage of maximum. (E) Serum level of TNFα and IL-6 in DSS-induced colitis mice model was determined by ELISA. The mean value of five mice ±SEM is presented. * *p* < 0.05 compared with DSS-treated group.

To elucidate whether the administration of chrysophanol blocks the histological deterioration by DSS-induced inflammation in colon tissue, isolated colons were sectioned and stained with hematoxylin and eosin (H&E). Tissues from the DSS group showed high shrinkage and destruction by severe inflammation, but tissues from the DSS group after oral administration of chrysophanol recovered in a dose-dependent manner (Figure 5A). Histological score also showed that chrysophanol administration ameliorates inflammatory manifestations in colon tissue (Figure 5B). Since chrysophanol showed the suppressive effect on nuclear translocation of p65 in HT-29 colon cells, we performed Western blot to understand whether oral administration of chrysophanol also inhibits the nuclear translocation of p65 in colon tissues. Colon tissues from the DSS group significantly exhibited increased p65 translocation into the nucleus but oral administration of chrysophanol reduced it compared to the DSS group in a dose-dependent manner (Figure 5C), which is consistent with the in vitro result. To investigate whether oral administration of chrysophanol attenuates the mRNA levels of inflammatory genes in colon tissue, gene expression associated with DSS-induced inflammation, such as *TNF-α, IL-1β, IL-8,* and *IL-6*, was checked by quantitative PCR analysis. As shown in Figure 5D, oral administration of chrysophanol significantly reduced the mRNA levels of these genes in a DSS-induced colitis model in a dose-dependent manner. Results from ELISA confirmed that chrysophanol suppressed the production of serum TNFα and IL-6 in the colitis model (Figure 5E). These data suggest that chrysophanol protects colon tissues from inflammatory destruction induced by DSS and mitigates the expression of pro-inflammatory genes in colons through the NF-κB signaling pathway.

### 2.6. Oral Administration of Chrysophanol Decreases the Expression of Effector Cytokines from Mesenteric Lymph Nodes

**Figure 6:**
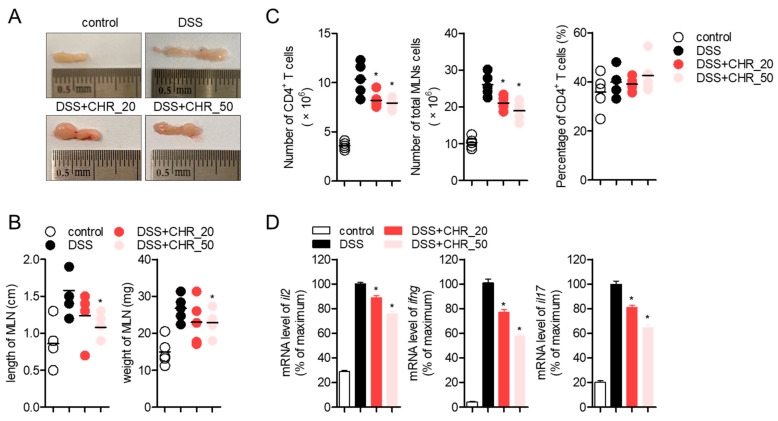
Oral administration of chrysophanol decreases the expression of effector cytokines from mesenteric lymph nodes. (**A**) The representative images of mesenteric lymph nodes from each mice group. (**B**) The length and weight of mesenteric lymph nodes from each mice group. (**C**) The number of CD4^+^ T cells, total mesenteric lymph nodes (MLNs) cells and percentage of CD4^+^ T cells in total MLNs cells. (**D**) The mRNA levels of the indicated genes on CD4^+^ T cells isolated from mesenteric lymph nodes were normalized with the levels of *GAPDH* and presented in percentage of maximum. The mean value of five mice ±SEM is presented. * *p* < 0.05 compared with DSS-treated group.

It has been previously reported that chrysophanol has a modulatory effect on T cell activity [20]. Since several studies have demonstrated that the activity of T cells is highly involved in the development of the DSS-induced colitis model [25,26,27], we examined whether oral administration of chrysophanol systemically ameliorates T cell activation in a DSS-induced colitis model. We first checked the mesenteric lymph nodes (MLNs) where several inflammatory cells, including T cells, are recruited to control colonic inflammation. Figure 6A shows that swelling in mesenteric lymph nodes (MLNs) by DSS-induced inflammation was significantly reduced by oral administration of chrysophanol in a dose-dependent manner. The length and weight of MLN were clearly downregulated by oral administration of chrysophanol (Figure 6B). To examine the regulatory effect of chrysophanol on T cells, CD4^+^ T cells were isolated from MLNs cells and further experiments were performed. As shown in Figure 6C, the number of CD4^+^ T cells and total MLNs cells were significantly reduced compared to DSS group but percentage of CD4^+^ T cells was not altered by oral administration of chrysophanol. Results from RT-PCR quantitative PCR analysis exhibited that mRNA levels of *IL-2*, *IFN*-γ, and *IL-17* were systemically mitigated by oral administration of chrysophanol on CD4^+^ T cells from MLNs. These results suggest that chrysophanol administration systemically decreases the activity of effector T cells in MLNs.

## 3. Discussion

**Figure 7:**
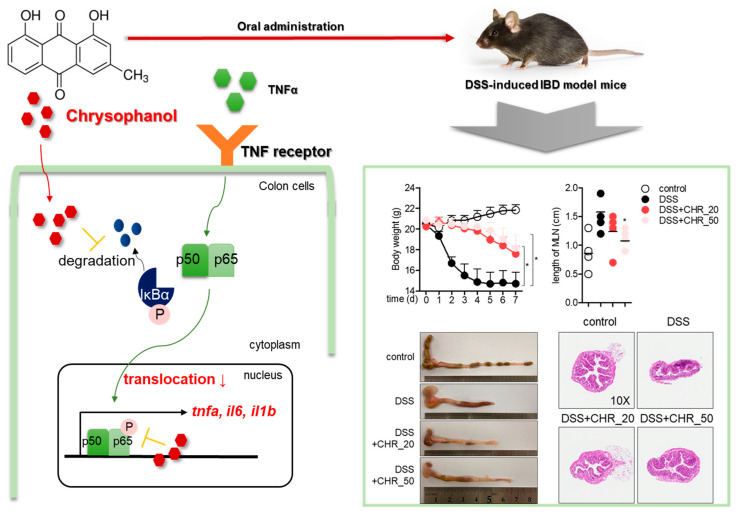
A schematic model for the protective effect of chrysophanol in the DSS-induced IBD model. * *p* < 0.05 compared with DSS-treated group.

In this study, we explored the modulatory effect of chrysophanol on the activity of intestinal epithelial cells in vitro and in a colitis model in vivo. It was found that chrysophanol has no negative effect on cytotoxicity up to 40 μM and does not lead to an apoptotic pathway in HT-29 colorectal cells. Quantitative PCR and Western blot analysis showed that pre-treatment with chrysophanol effectively reduced the mRNA levels of pro-inflammatory cytokines through the NF-κB and MAPK pathways under TNF-α stimulation. Oral administration of chrysophanol revealed a protective effect in a DSS-induced IBD model by downregulating inflammation in colon tissue and regulating the activity of effector Th1/Th17 cells in vivo. A schematic model is shown in Figure 7.

HT-29 cells, intestinal epithelial cells have been widely used to study colitis and in intestinal research [28]. In particular, stimulation of HT-29 cells with TNF-α promotes inflammatory conditions where pro-inflammatory cytokines and chemokines are involved in [29]. IL-8, a pro-inflammatory cytokine produced from HT-29 by stimulation with TNF-α, is a chemokine that acts as a chemoattractant to recruit neutrophils and basophils to the inflammatory lesion [30]. IL-1β and IL-6 have been elucidated as potent pro-inflammatory cytokines that stimulate several immune cells in colitis conditions [31]. The DSS-induced IBD model was first established for studying experimental colitis and intestinal inflammation and is the most widely used animal model for inflammation research [32]. The current study demonstrated that chrysophanol pretreatment effectively suppressed the mRNA levels of TNF-α, IL-8, IL-1β, and IL-6 in TNF-α-stimulated HT-29 cells in a dose-dependent and time-dependent manner (Figure 2). In addition, consistent in vivo results were also revealed in the DSS-induced IBD model that oral administration of chrysophanol ameliorated IBD manifestation by regulating colonic inflammation (Figure 4 and Figure 5). Our study is the first to report the regulatory effect of chrysophanol on intestinal inflammation in HT-29 cells and an IBD model.

The role of effector T cells in IBD has been examined, and differentiation into Th1/Th17 cells is critical for the pathogenesis of IBD [33]. To be differentiated into effector T cells, T cell priming by antigen-presenting cells and TCR-mediated stimulation are essential in draining lymph nodes. In Crohn’s disease, autoreactive T cells primed by self-antigens show excessive activity in intestinal inflammation that triggers chronic inflammation in the colon. Therefore, modulation of T cell activity as well as colorectal cell activity would be beneficial as a development strategy for IBD therapeutics. In a previous study, we found that chrysophanol has a regulatory effect on T cell activation through downregulation of CD40L expression, which is critical for T-antigen presenting cell conjugation [20]. We also investigated the inhibitory effect of chrysophanol on the activity of colorectal cells stimulated with TNF-α. Results from animal experiments using the DSS-induced IBD model clearly showed that the mRNA levels of effector cytokines from Th1/Th17 cells were reduced in mice administered chrysophanol (Figure 6). These results suggest the potential of chrysophanol as a novel therapeutic agent for IBD, which is a T-cell mediated disease. Further studies should include the clear underlying mechanism of whether chrysophanol is mediated by T cell differentiation into effector T cells.

Recent investigations in gastroenterology have focused how gut microbiota affects to the pathogenesis of colitis [34,35]. There have been some experimental trials to explore the relationship between oral administration of chrysophanol and gut microbiota in biochemical and pharmacological methods. Analysis of the metabolites of anthraquinones including chrysophanol by human intestinal bacteria has been firstly reported [36]. The involvement of chrysophanol in diarrhea and gut microbiota has been also explored by analyzing the role of tryptophan-metabolizing microbiota [37]. A recent report has shown that anthraquinone including chrysophanol suppresses type 2 diabetes by regulating the gut microbiota and colon inflammation [38]. In particular, this literature has exhibited that a mixture of anthraquinone containing chrysophanol ameliorates the intestinal inflammation by changing the diversity of its microbiota and by improving guts as “healthy” gut in type 2 diabetes rats. These previous investigations assume that chrysophanol may have a beneficial effect on gut microbiota such that it enhances probiotic microbiota in the intestinal inflammation condition. Further studies should focus on the bioactivity of chrysophanol on the gut microbiota, how the chrysophanol connects with IBD and gut microbiota.

## 4. Materials and Methods

### 4.1. Cells

HT-29 colorectal cells (KCLB number: 30038) were purchased from the Korean Cell Line Bank (Seoul, Korea) and cultured in Dulbecco’s Modified Eagle Medium (DMEM) medium (Welgene, Gyeongsan, Korea) supplemented with 1× streptomycin and penicillin G, 10% fetal bovine serum, and L-glutamine (2 mM). Cells were passaged five to eight times and maintained at 37 °C in a humidified incubator containing 5% CO_2_.

### 4.2. Mice

Six-to eight-week-old female C57BL/6J mice were obtained from Samtako Bio (Osan, Korea) and housed under specific pathogen-free (SPF) conditions. All experiments were approved by the Animal Care and Use Committee of the College of Pharmacy, Keimyung University (approval number: KM2020-004).

### 4.3. Isolation of Chrysophanol from Rumex crispus L.

Chysophanol was isolated from dried leaves of *R. crispus* L as reported [10]. Dried leaves of *R. crispus* L (500 g) were extracted three times with 70% ethanol for 20 h under room temperature. The EtOH extract (115 g) was suspended in H_2_O (500 mL) and then partitioned with n-hexane (500 mL) and EtOAc (500 mL). Among the n-hexene (2.25 g) and EtOAc fraction (13.55 g), chrysophanol was purified by repeated open column chromatography using silica gel (6.5 × 60 cm; 70–230 mesh). The isolated chrysophanol was analyzed by nuclear magnetic resonance (NMR) spectroscopic, and spectral data were identified as chrysophanol by comparison with the published literature [39]. Isolated chrysophanol was dissolved in dimethyl sulfoxide (DMSO) at 40 mM as stock and treated after calculation of volume depending on the concentration into medium.

### 4.4. Reagents and Antibodies

Methyl thiazol tetrazolium (MTT) powder and radioimmunoprecipitation assay (RIPA) buffer were obtained from Sigma Chemical Co. (St. Louis, MO, USA). Annexin V and caspase 3/7 staining reagents for the IncuCyte^®^ cell imaging system were purchased from Essen Bio (Ann Arbor, MI, USA). Human TNF-α recombinant protein was obtained from PeproTech EC Ltd. (London, UK). Dextran sulfate sodium (DSS) was purchased from MP biomedicals (Irvine, CA, USA). Polyvinylidene fluoride (PVDF) membranes for Western blotting, enhanced chemiluminescence (ECL) Western blot detection reagent, and NE-PER kit were obtained from Thermo Scientific (Rockford, IL, USA). Antibodies against p65 (Cat. #4764), LaminB (Cat. #13435), IκBα (Cat. #9242), phosphorylated IκBα (Cat. #2859), ERK (Cat. #9102), p38 (Cat. #9212), phosphorylated p38 (Cat. #9211), JNK (Cat. #9252), and phosphorylated JNK (Cat. #9251) were purchased from Cell Signaling Technology (Beverly, MA, USA). Anti-β-actin (Cat. #sc-47778) and anti-phosphorylated ERK (Cat. #sc-7383) were obtained from Santa Cruz Biotechnology (Dallas, TX, USA).

### 4.5. MTT Viability Assay

HT-29 cells (1 × 10^4^/well, 96-well plate) were treated with the indicated concentration (0–40 μM) of chrysophanol for 24 h. Cells were treated with MTT (500 μg/mL) for 2 h and supernatants were discarded. Formazan crystals at the bottom of the wells were dissolved with 170 μL of dimethyl sulfoxide and the plate was read at 540 nm to obtain the OD values of each well. Cell viability was calculated using the OD value of control cells and presented as a percentage of the control.

### 4.6. Determination of AnnexinV and Caspase3/7 Expression

The expression of AnnexinV and Caspase3/7 was determined using the IncuCyte^®^ imaging system. HT-29 cells were seeded (1 × 10^4^/well, 96-well plate) and stained with 1 × AnnexinV and Caspase3/7 staining reagents. Cells were incubated with the indicated concentrations of chrysophanol (0–40 μM) for 24 h and fluorescence was scanned using the IncuCyte^®^ imaging system. The intensity of AnnexinV and Caspase3/7 was calculated as % of the control and presented as a bar graph.

### 4.7. Quantitative Realtime PCR Analysis

To measure the mRNA levels of the indicated genes in HT-29 cells, colon tissues, or mesenteric lymph nodes, total RNA was isolated using TRIZOL reagent and reverse transcription was performed using RT PreMix (Enzynomics, Daejeon, Korea). The DNA Engine Opticon 1 continuous fluorescence detection system (MJ Research, Waltham, MA, USA) with SYBR Premix Ex Taq (Takara, Japan) was used for quantitative real-time PCR analysis. The total reaction volume was 10 μL, containing 1 μL of cDNA or control and gene-specific primers (Table 1). Each PCR reaction was performed using the following conditions: 95 °C for 30 s, 60 °C for 30 s, 72 °C for 30 s, and plate read (detection of fluorescent product) for 40 cycles, followed by 7 min of extension at 72 °C. A melting curve was drawn to characterize the dsDNA product by gradually increasing the temperature (0.2 °C/s) from 60 °C to 95 °C with collection of fluorescence data at 0.2 °C intervals. The mRNA levels of cytokines normalized to *GAPDH* were presented as % of the maximum. The maximum percentage was calculated using the following equation: % of maximum = 2^−ΔΔCT^ × 100, where ΔΔCT = (CT_target_ − CT_gapdh_) at maximum (CT_target_ − CT_gapdh_). Here, time x represents any time point and time 0 represents the 1× expression of genes indicated in control cells normalized to *GAPDH*.

### 4.8. Western Blot Analysis

HT-29 cells incubated with the indicated conditions were collected and lysed for Western blotting using a RIPA buffer. After lysis for 20 min on ice, cells were centrifuged at 14,000 rpm for 20 min at 4 °C, and supernatants were harvested for further analysis. For the separation of nuclear extracts from whole lysates, the NE-PER kit was used with the manufacturer’s instructions. Approximately 30 to 50 μg of lysate was loaded on 8–12% sodium dodecyl sulfate (SDS)-polyacrylamide gel electrophoresis gels and run for protein separation. Proteins were transferred onto PVDF membranes which were blocked with blocking buffer (5% skim milk) for 1 h. After rinsing with Tris-buffered saline containing 0.1% Tween 20 (TBS-T), membranes were incubated with primary antibodies in 1% skim milk in TBS-T overnight at 4 °C (dilution factor was 1:1000). After incubation, excess primary antibodies were removed with five washes with TBS-T and membranes were incubated with 0.1 μg/mL peroxidase-labeled secondary antibodies (against rabbit or mouse) for 1.5 h. After five washes with TBS-T, specific bands were visualized using ECL detection reagents (Thermo Fisher Scientific, Waltham, MA, USA) and an ImageQuant LAS 4000 (GE Healthcare, Chicago, IL, USA). Every detected band was normalized to β-actin (cytosolic extracts) and LaminB (nuclear extracts). Total proteins of the MAPK pathway were used for normalization of phosphorylation levels of the MAPK pathway.

### 4.9. Induction of IBD Model Using DSS

The IBD model was induced using DSS in drinking water by slightly modifying the following references [32,40]. According to the sample size calculation (alpha = 0.05, power = 0.8), twenty mice were separated into four groups: control mice group with fresh water, DSS mice group fed drinking water containing 2.5% DSS for 7 days, DSS + CHR_20 mice group fed drinking water containing 2.5% DSS and daily oral administration of 20 mg/kg chrysophanol for 7 days, and DSS + CHR_50 mice fed drinking water containing 2.5% DSS and daily oral administration of 50 mg/kg chrysophanol for 7 days (*n* = 5 mice/group). Changes in body weight, disease activity, and stool score were monitored every day.

### 4.10. Determination of Disease Activity Index

To evaluate the clinical progression of colitis, the disease activity index was measured for 7 days by following a reported reference [41]. The disease activity index contains the score of relative weight loss, consistency of stool, and bleeding around the anus and stool. The scoring definition was as follows: weight loss: 0 (no loss), 1 (1–5%), 2 (5–10%), 3 (10–20%), and 4 (more than 20%); stool form: 0 (normal), 2 (loose stool), and 4 (diarrhea); and bleeding: 0 (no blood), 1 (Hemoccult positive), 2 (Hemoccult positive and visual pellet bleeding), and 4 (bleeding around anus).

### 4.11. Histological Analysis with H&E Staining

After mice were sacrificed, the colons were harvested and subjected to histopathological analysis. The removed colons were fixed in 10% paraformaldehyde and embedded in paraffin. Embedded tissues were sliced (5-μm-thick sections) and deparaffinized. Deparaffinized tissues were stained with hematoxylin and eosin (H&E) for histological analysis. To evaluate the histological score from each group, crypt architecture and degree of inflammatory cell infiltration was monitored by following the reported reference [42]. Crypt architecture: normal, 0 to severe destruction with entire crypt loss, 3; degree of inflammatory cell infiltration, normal; 0 to severe infiltration of immune cells; 3.

### 4.12. Purification of CD4^+^ T Cells from Mesenteric Lymph Nodes

From collected MLNs, CD4^+^ T cells were isolated by the magnetic-activated cell sorting (MACS) separation system (Miltenyi Biotec. Bergisch Gladbach, Germany) as in the references [43,44]. After isolation of CD4^+^ T cells, a percentage of CD4^+^ T cells in total cells from MLNs was calculated by counting the cells.

### 4.13. Statistics

One-way ANOVA was used to assess significance (*p* value). Mean values ±SEM were calculated from the acquired data of three independent experiments performed on separate days or five independent mice for animal experiments and are presented in graphs with *p* values (*). * represents significant differences compared with the indicated two groups at *p* < 0.05.

## Figures and Tables

**Table 1:** Primer sequence used for the quantitative real-time PCR analysis.

Gene Name	Forward Sequence (5′-3′)	Reverse Sequence (5′-3′)
human *TNFα*	CCT ACC AGA CCA AGG TCA AC	AGG GGG TAA TAA AGG GAT TG
human *IL-1β*	GGA TAT GGA GCA ACA AGT GG	ATG TAC CAG TTG GGG AAC TG
human *IL-8*	GTG CAG TTT TGC CAA GGA GT	TTA TGA ATT CTC AGC CCT CTT CAA AAA
human *GAPDH*	CGG AGT CAA CGG ATT TGG TCG TAT	AGC CTT CTC CAT GGT GGT GAA GAC
mouse *TNFα*	GGC AGG TCT ACT TTG GAG TCA TTG C	ACA TTC GAG GCT CCA GTG AAT TCG G
mouse *IL-1β*	ATA ACC TGC TGG TGT GTG AC	AGG TGC TGA TGT ACC AGT TG
mouse *IL-8*	ATG GCT GCT CAA GGC TGG TC	AGG CTT TTC ATG CTC AAC ACT AT
mouse *IL-6*	CCG GAG AGG AGA CTT CAC AG	GGA AAT TGG GGT AGG AAG GA
mouse *IL-2*	TGA GCA GGA TGG AGA ATT ACA GG	GTC CAA GTT CAT CTT CTA GGC AC
mouse *IFNγ*	TCA AGT GGC ATA GAT GTG GAA GAA	TGG CTC TGC AGG ATT TTC ATG
mouse *IL-17*	TCC CCT CTG TCA TCT GGG AAG	CTC GAC CCT GAA AGT GAA GG
mouse *GAPDH*	GCA CAG TCA AGG CCG AGA AT	GCC TTC TCC ATG GTG GTG AA

## Data Availability

The data presented in this study are available on request from the corresponding author.

## Sample Availability

Samples of the compounds are not available from the authors.
